# Evaluating knowledge and awareness of 3D design and printing among dental students in Saudi Arabia: a cross-sectional study

**DOI:** 10.3389/fdmed.2024.1466393

**Published:** 2024-11-25

**Authors:** Hamdan Alamri, Falah R. Alshammari, Abdullah Bin Rahmah, Mohammed I. Alsaif, Farah Almutairi, Hissah Alolaywi, Aroub Altariqi, Sarah Alotaibi, Rahaf Almutairi, Hossam Almadhoon, Hani S. AlMoharib

**Affiliations:** ^1^ Community Dentistry and Oral Epidemiology Department, College of Dentistry, Qassim University, Buraydah, Saudi Arabia; ^2^ Dental Public Health and Community Dentistry, College of Dentistry, University of Ha’il, Ha’il, Saudi Arabia; ^3^ Department of Periodontics and Community Dentistry, College of Dentistry, King Saud University, Riyadh, Saudi Arabia; ^4^ College of Dentistry, Majmaah University, Al-Majmaah, Saudi Arabia; ^5^ Faculty of Dentistry, Alazhar University—Gaza, Gaza Strip, Palestine

**Keywords:** three-dimensional printing, 3D design, knowledge, dental students, Saudi Arabia

## Abstract

**Introduction:**

As 3D design and printing technology gains popularity, there remains limited evidence on dental students' perceptions in Saudi Arabia regarding its use. This study aims to assess the knowledge and awareness of dental students in Saudi Arabia about 3D design and printing technology.

**Methods:**

A cross-sectional questionnaire-based study was conducted among dental students in their third, fourth, and fifth years at multiple universities in Saudi Arabia between February and October 2023. A validated self-administered questionnaire with 15 close-ended items, including demographic and knowledge-related questions about 3D design and printing, was used. Statistical analysis was performed using Chi-square and Fisher exact tests to identify factors associated with knowledge and awareness levels.

**Results:**

A total of 374 dental students participated in the study, with 63.1% being female students. Of the participants, 40.4% identified the cost of equipment as the primary barrier to 3D printing usage in dentistry in Saudi Arabia. The majority (94.4%) recognized the advantages of 3D models for implant placement. Awareness of 3D printing utilization in the field was reported by 69.3% of participants, and 74.3% acknowledged its increasing popularity. Furthermore, 73.8% of participants expressed confidence in the biocompatibility and safety of 3D printed materials. A significant percentage (64.0%) were aware of 3D printing's role in creating Invisalign aligners, and 42.4% believed in the value of 3D printed drill guides for root canal treatment. The study found statistically significant regional differences (*p* < 0.05) across all questions. Participants primarily relied on colleges (64.8%) as their source of information, and a high proportion (82.4%) expressed interest in further exploring the usage of 3D printing in dentistry.

**Conclusion:**

Our study found that students' knowledge and awareness in Saudi Arabia are generally satisfactory. Integrating 3D printing into dental curricula and providing workshops is crucial to meet dental students' interest in exploring its usage and equipping them for its future implementation.

## 1 Introduction

The appearance of 3D printing technology has revolutionized the dentistry discipline, offering a multitude of benefits for both dental professionals and patients (1–3). This technology facilitates the creation of highly precise, customized dental prostheses, models, and other devices that were previously unattainable using traditional manufacturing methods (4, 5). As a result, 3D printing is increasingly becoming an essential tool in the dental industry, permitting faster, more efficient, and more accurate dental treatments (6).

3D printed structural models play a crucial role in simulating and planning dental surgical procedures (7). For instance, in implantology, the integration of CAD-CAM technologies, alongside CBCT, allowed for the precise placement of implants with minimal invasiveness (8). This is achieved through the production of tailored surgical guides based on detailed digital scans and virtual treatment plans, thereby enhancing surgical accuracy and reducing patient discomfort. In orthodontics, 3D printing offers personalized solutions such as aligners and brackets that adapt to individual treatment needs (9). Similarly, prosthodontics benefits from this technology as dentists can create customized crowns, bridges, and dentures with unprecedented precision, leading to improved fit and functionality (10). Further, the use of biocompatible 3D printing materials is opening new avenues for research and development, such as the creation of scaffolds for tissue engineering and complex anatomical models for educational purposes (5). Nevertheless, challenges remain, including the high costs of materials, processing expenses, and labor-intensive post-processing (5).

According to Suganna et al., knowledge and awareness of 3D printing technology could have a big impact on dental education, research, and practice in the future, potentially leading to the creation of educational curricula, clinical recommendations, and technological advancements that could raise the standard and accessibility of dental treatment (6). Recently, attention to the applications of 3D printing in dentistry has grown, both in research and in practice (7). However, there is still much to learn about the potential of this technology, and more research is needed to fully comprehend its capabilities and limitations. Meanwhile, it has been found that fewer studies discuss advances in 3D printing, nor do many show concern for the assessment of the knowledge level (11).

In the context of Saudi Arabia, further research is necessary to evaluate the knowledge and awareness levels of dental students regarding 3D design and printing technologies in dentistry (12). Such assessments are vital for understanding how students perceive and interact with these advanced technologies, enabling dental colleges to develop targeted interventions to address any gaps in knowledge and cater to students' needs. This study aims to assess the current level of knowledge and awareness among dental students in Saudi Arabia regarding 3D design and printing technology.

## 2 Methods

The study adhered to the Strengthening the Reporting of Observational Studies in Epidemiology (STROBE) guidelines (13). Approval for the study proposal (MUREC- lan.29/CoNt-2022/5-2) was granted by the Majmaah University for Research Ethics Committee (HA-01-R-088).

### 2.1 Study design and setting

The current cross-sectional study took place at multiple universities in Saudi Arabia from 7 February to 25 October 2023. We chose the dental schools participating in the study based on their feasibility for data collection. A detailed list of the included dental schools can be found in Supplementary File 1.

### 2.2 Study participants and data collection

Dental students in their third, fourth and fifth years of study from the selected dental schools constituted our targeted participants. These students were approached by a member of the study team through social media apps, such as WhatsApp, Telegram, and X (previously, Twitter), with posts outlining the details of the study and a link to the survey. The participants' information was kept confidential, and their informed consent was sought prior to the commencement of the study. Potential respondents had six weeks to complete the survey after providing their consent. Responses to the survey were collected online through the Google Forms tool in English and stored in Microsoft Excel using Google Surveys.

### 2.3 Data sampling techniques

A convenience sampling method was employed to gather participant responses. The required sample size of 370 was calculated using Epi Info 7, considering a confidence limit of 5%, an anticipated frequency of 50%, an acceptable margin of error of 0.05, a design effect of 1.0, and clusters of 1.0.

### 2.4 Study instrument

The study used a validated self-administered questionnaire that was adopted and developed by previously published similar studies (6, 7). The developed tool was reviewed by experts from King Saud University's College of Dentistry, and a pilot survey was conducted to assess practicality and identify any errors before data collection. No modifications were made to the original questionnaire. The questionnaire comprised 14 items with closed-ended questions divided into two segments. The first section gathered the demographic variables (gender, region, university name, academic level) of the respondents. The second section contained questions designed to assess the knowledge and awareness in relation to 3D design and printing of undergraduate dental students in Saudi Arabia.

### 2.5 Statistical analysis

The collected quantitative data was coded and cleaned using Microsoft Excel, and statistical analysis was performed using R software version 4.2.2. Categorical data was presented as numbers with corresponding percentages. Chi-square test and Fisher exact test were employed to determine factors associated with knowledge and awareness of 3D design and printing among Saudi dental students. A significance level of *p* < 0.05 was considered statistically significant.

## 3 Results

### 3.1 Sociodemographic data

A total of 374 dental students participated in the study, with a response rate of 81.3%. Higher participation was seen among female students (63.1%) compared to male students (36.9%). In terms of region, the highest representation was from the central region (32.9%), followed by the eastern region (30.7%). The smallest proportion was seen from the southern region (3.5%). Regarding academic level, the largest cohort was in the fifth year (44.7%), followed by the third year (29.7%) and the fourth year (25.7%) (see Table 1).

**Table 1:** Sociodemographic data of the study participants.

Basic characteristics		*N*	%
Gender	Male	138	36.9%
	Female	236	63.1%
Region	Central	123	32.9%
	Eastern	115	30.7%
	Western	53	14.2%
	Northern	70	18.7%
	Southern	13	3.5%
Academic level	Third year	111	29.7%
	Fourth year	96	25.7%
	Fifth year	167	44.7%

### 3.2 Knowledge of participants towards 3D design and printing in dentistry

**Figure 1:**
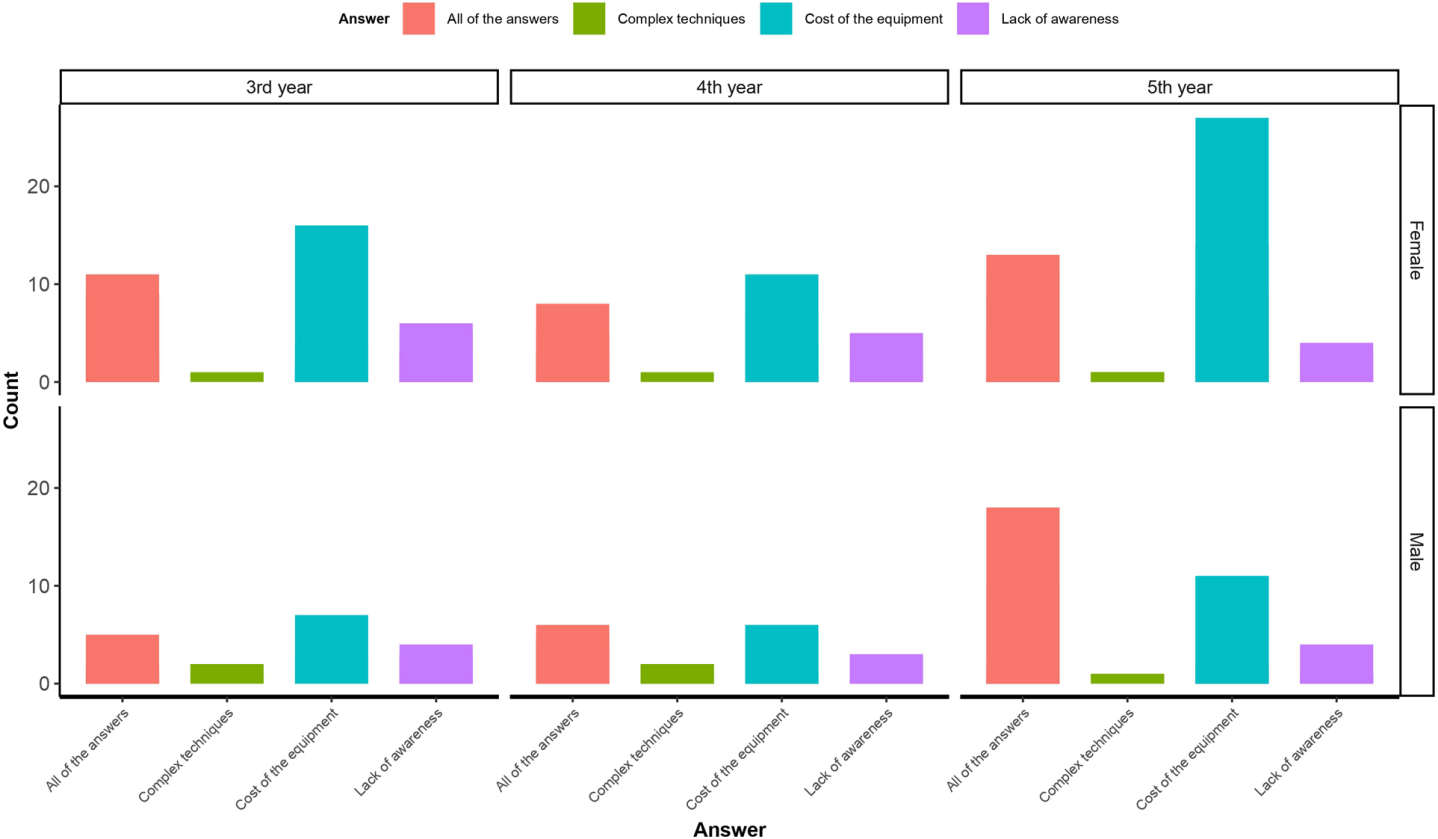
Dental students' perspectives on why 3D printing is not widely used in Saudi Arabian dentistry compared to other countries.

Frequencies of participants' answers for each question and the differences based on sociodemographic factors regarding knowledge about 3D design and printing in dentistry are presented in Table 2. Participants were also asked about the reasons why 3D printing is not widely used in Saudi Arabian dentistry compared to other countries (Figure 1). Of the respondents, 40.4% indicated the cost of equipment as the main barrier, while only 16.6% acknowledged lack of awareness and 4.0% referred to technique complexities as the sole reasons. The results also found statistically significant differences between participants based on education level and geographic region (*p* < 0.05). Regarding the benefits of performing mock surgery using 3D printed models, participants indicated that increased accuracy and predictability, reduced operating time, and enhanced patient safety were collectively the most frequent responses (61.8%), with the region being the only statistically significant factor (*p* < 0.05). Most participants (94.4%) recognized the advantages of 3D models for correct implant placement (most accurate position and least complicated procedure).

**Table 2:** Participants' knowledge/perspectives based on sociodemographic factors such as gender, region, and academic level.

Questions	What do you think might be the reason why 3D printing is not widely used in Saudi Arabia in the field of dentistry when compared to other countries?^$^				What do you think might be the benefits of performing mock surgery using 3D printed models in cases of complex craniofacial fractures and surgeries?				3D printed implant guides mean that the placement of implants is the	
Answers	Cost of the equipment	Lack of awareness	Complex techniques	All of the above	Surgery becomes more accurate and predictable	Less time spent on the operating table	Patient's safety is ensured	All of the above^£^	Most accurate position and least complicated procedure^£^	Least accurate and more complicated procedure
	*n* = 151 (40.4%)	*n* = 62 (16.6%)	*n* = 15 (4.0%)	*n* = 146 (39.0%)	*n* = 85 (22.7%)	*n* = 46 (12.3%)	*n* = 12 (3.2%)	*n* = 231 (61.8%)	*n* = 353 (94.4%)	*n* = 21 (5.6%)
Gender (%)										
Male	48 (31.8)	25 (40.3)	10 (66.7)	55 (37.7)	30 (35.3)	16 (34.8)	4 (33.3)	88 (38.1)	133 (37.7)	5 (23.8)
Female	103 (68.2)	37 (59.7)	5 (33.3)	91 (62.3)	55 (64.7)	30 (65.2)	8 (66.7)	143 (61.9)	220 (62.3)	16 (76.2)
*p-* value	0.051				0.942				0.295	
Level (%)										
3rd year	37 (24.5)	28 (45.2)	4 (26.7)	42 (28.8)	24 (28.2)	14 (30.4)	6 (50.0)	67 (29.0)	103 (29.2)	8 (38.1)
4th year	36 (23.8)	21 (33.9)	6 (40.0)	33 (22.6)	22 (25.9)	17 (37.0)	4 (33.3)	53 (22.9)	90 (25.5)	6 (28.6)
5th year	78 (51.7)	13 (21.0)	5 (33.3)	71 (48.6)	39 (45.9)	15 (32.6)	2 (16.7)	111 (48.1)	160 (45.3)	7 (33.3)
*p-*value	0.002*				0.161				0.537	
Region (%)										
Central	40 (26.5)	22 (35.5)	4 (26.7)	57 (39.0)	31 (36.5)	16 (34.8)	2 (16.7)	74 (32.0)	122 (34.6)	1 (4.8)
Eastern	78 (51.7)	13 (21.0)	4 (26.7)	20 (13.7)	26 (30.6)	7 (15.2)	1 (8.3)	81 (35.1)	112 (31.7)	3 (14.3)
Northern	17 (11.3)	9 (14.5)	5 (33.3)	39 (26.7)	13 (15.3)	6 (13.0)	2 (16.7)	49 (21.2)	64 (18.1)	6 (28.6)
Southern	3 (2.0)	4 (6.5)	0 (0.0)	6 (4.1)	4 (4.7)	2 (4.3)	2 (16.7)	5 (2.2)	8 (2.3)	5 (23.8)
Western	13 (8.6)	14 (22.6)	2 (13.3)	24 (16.4)	11 (12.9)	15 (32.6)	5 (41.7)	22 (9.5)	47 (13.3)	6 (28.6)
*p-*value	<0.001*				<0.001*				<0.001*	

* Statistically significant factor.

### 3.3 The awareness of dental students regarding 3D design and printing in dentistry

**Figure 2:**
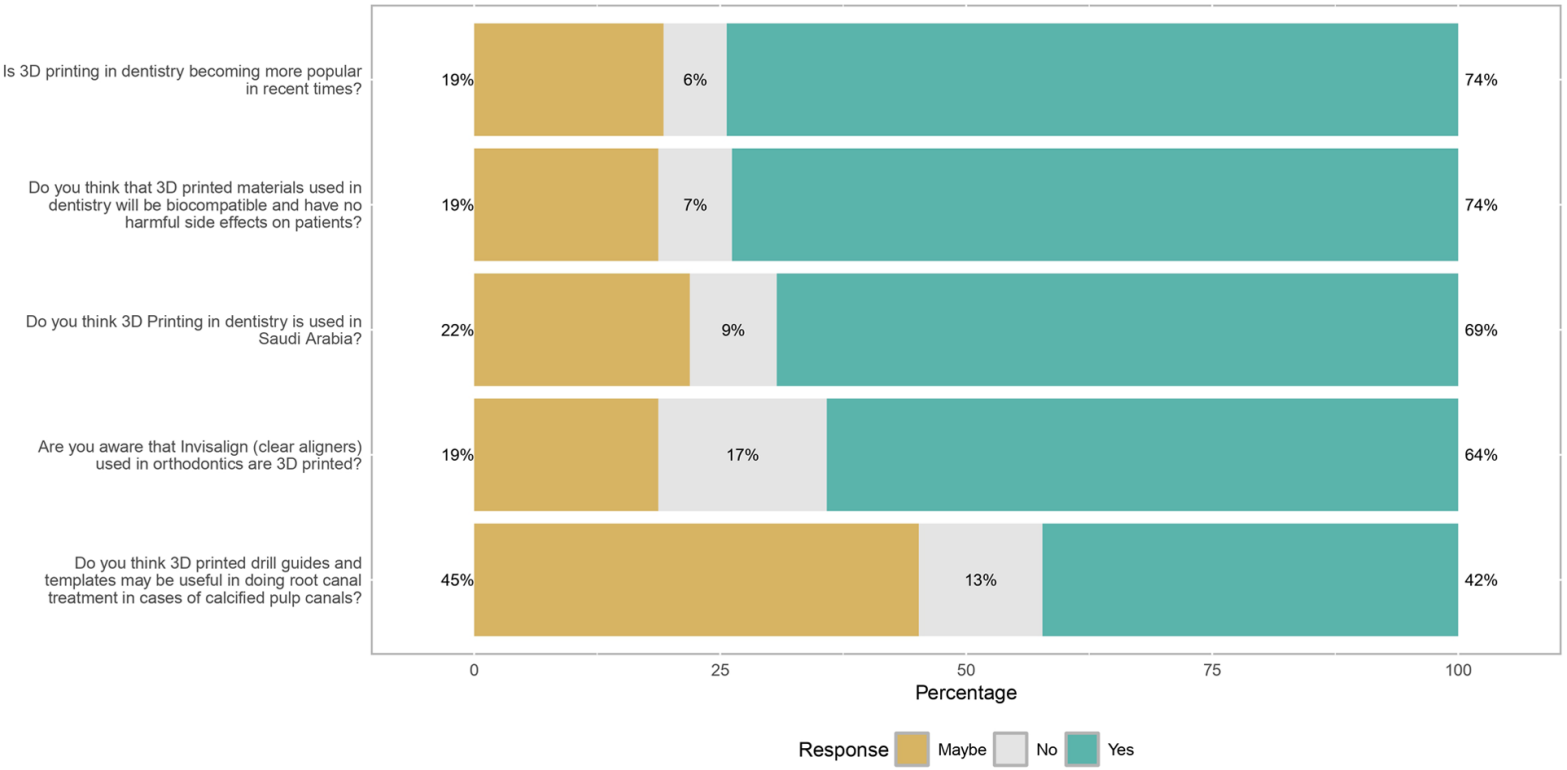
Overall awareness of dental students towards 3D design and printing (*n* = 374).

**Figure 3:**
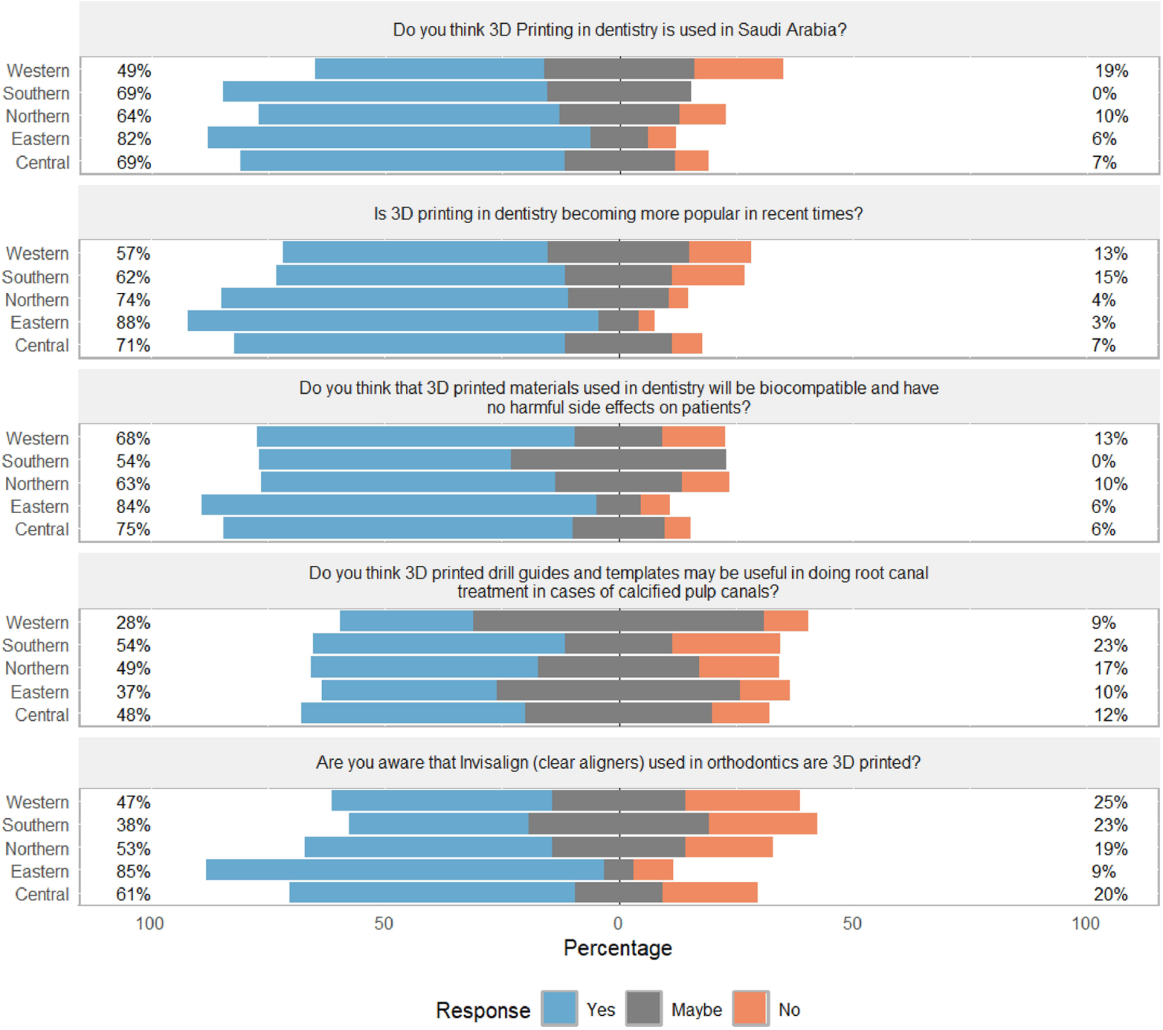
Awareness of dental students towards 3D design and printing by geographic region.

Frequencies of participants' answers for each question and the differences based on sociodemographic factors regarding awareness of 3D design and printing in dentistry are presented in Table 3. Among participants, 69.3% and 74.3% were aware that 3D printing is utilized in the field in Saudi Arabia and is becoming increasingly popular, respectively (see Figure 2). For both questions, there was a significant difference based on study level and region (*p* < 0.05) (see Table 3). The majority (73.8%) also expressed confidence that 3D printed materials are biocompatible and not harmful to patients. Additionally, 64.0% of participants were aware of 3D printing's role in creating Invisalign clear aligners, and 42.4% believed that 3D printed drill guides and templates can be valuable for performing root canal treatment in cases of calcified pulp canals (see Figure 3 and Table 3).

**Table 3:** The participants' awareness based on sociodemographic factors such as gender, region, and academic level.

Variables	Do you think 3D Printing in dentistry is used in Saudi Arabia?			Is 3D printing in dentistry becoming more popular in recent times?			Do you think that 3D printed materials used in dentistry will be biocompatible and have no harmful side effects on patients?			Do you think 3D printed drill guides and templates may be useful in doing root canal treatment in cases of calcified pulp canals?			Are you aware that Invisalign (clear aligners) used in orthodontics are 3D printed?		
Answers	Yes *n* = 259 (69.3%)	Maybe *n* = 82 (21.9%)	No *n* = 33 (8.8%)	Yes *n* = 278 (74.3%)	Maybe *n* = 72 (19.3%)	No *n* = 24 (6.4%)	Yes *n* = 276 (73.8%)	Maybe *n* = 70 (18.7%)	No *n* = 28 (7.5%)	Yes *n* = 158 (42.2%)	Maybe *n* = 169 (45.2%)	No *n* = 47 (12.6%)	Yes *n* = 240 (64.2%)	Maybe *n* = 70 (18.7%)	No *n* = 64 (17.1%)
Gender (%)															
Male	97 (37.5)	27 (32.9)	14 (42.4)	97 (34.9)	28 (38.9)	13 (54.2)	99 (35.9)	26 (37.1)	13 (46.4)	61 (38.6)	60 (35.5)	17 (36.2)	83 (34.6)	23 (32.9)	32 (50.0)
Female	162 (62.5)	55 (67.1)	19 (57.6)	181 (65.1)	44 (61.1)	11 (45.8)	177 (64.1)	44 (62.9)	15 (53.6)	97 (61.4)	109 (64.5)	30 (63.8)	157 (65.4)	47 (67.1)	32 (50.0)
*p*-value	0.6			0.159			0.543			0.839			0.056		
Level (%)															
3rd year	63 (24.3)	36 (43.9)	12 (36.4)	75 (27.0)	28 (38.9)	8 (33.3)	79 (28.6)	24 (34.3)	8 (28.6)	45 (28.5)	55 (32.5)	11 (23.4)	61 (25.4)	27 (38.6)	23 (35.9)
4th year	60 (23.2)	25 (30.5)	11 (33.3)	62 (22.3)	23 (31.9)	11 (45.8)	67 (24.3)	20 (28.6)	9 (32.1)	45 (28.5)	36 (21.3)	15 (31.9)	59 (24.6)	18 (25.7)	19 (29.7)
5th year	136 (52.5)	21 (25.6)	10 (30.3)	141 (50.7)	21 (29.2)	5 (20.8)	130 (47.1)	26 (37.1)	11 (39.3)	68 (43.0)	78 (46.2)	21 (44.7)	120 (50.0)	25 (35.7)	22 (34.4)
*p*- value	<0.001*			0.001*			0.567			0.433			0.061		
Region (%)															
Central	85 (32.8)	29 (35.4)	9 (27.3)	87 (31.3)	28 (38.9)	8 (33.3)	92 (33.3)	24 (34.3)	7 (25.0)	59 (37.3)	49 (29.0)	15 (31.9)	75 (31.2)	23 (32.9)	25 (39.1)
Eastern	94 (36.3)	14 (17.1)	7 (21.2)	101 (36.3)	10 (13.9)	4 (16.7)	97 (35.1)	11 (15.7)	7 (25.0)	43 (27.2)	60 (35.5)	12 (25.5)	98 (40.8)	7 (10.0)	10 (15.6)
Northern	45 (17.4)	18 (22.0)	7 (21.2)	52 (18.7)	15 (20.8)	3 (12.5)	44 (15.9)	19 (27.1)	7 (25.0)	34 (21.5)	24 (14.2)	12 (25.5)	37 (15.4)	20 (28.6)	13 (20.3)
Southern	9 (3.5)	4 (4.9)	0 (0.0)	8 (2.9)	3 (4.2)	2 (8.3)	7 (2.5)	6 (8.6)	0 (0.0)	7 (4.4)	3 (1.8)	3 (6.4)	5 (2.1)	5 (7.1)	3 (4.7)
Western	26 (10.0)	17 (20.7)	10 (30.3)	30 (10.8)	16 (22.2)	7 (29.2)	36 (13.0)	10 (14.3)	7 (25.0)	15 (9.5)	33 (19.5)	5 (10.6)	25 (10.4)	15 (21.4)	13 (20.3)
*p-*value	0.003*			0.002*			0.006*			0.031*			<0.001*		

* Statistically significant factor.

### 3.4 Sources of information and interest in 3D printing in dentistry

**Figure 4:**
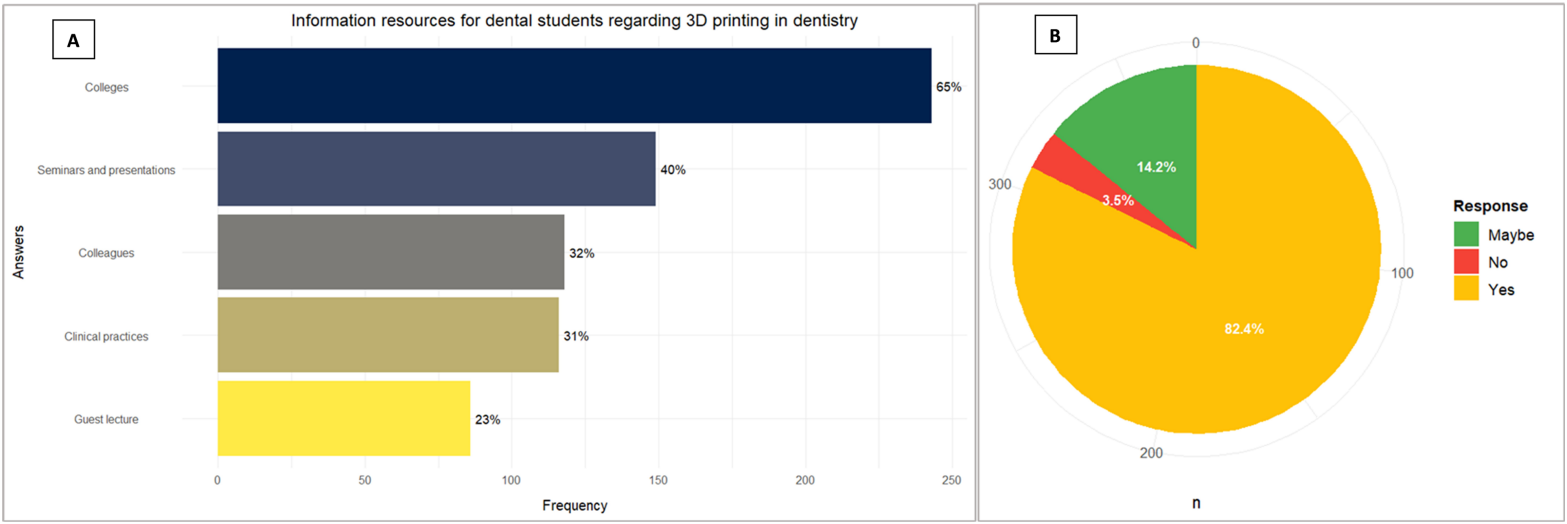
**(A)** Source of information through which dental students got to know about 3D printing in dentistry (multiple answers); **(B)** Dental students' interest in further exploring applications of 3D printing in dentistry.

Participants indicated that their primary sources of information about 3D printing in dentistry were colleges (64.8%), seminars and presentations (39.7%), and colleagues (31.5%) (see Figure 4). Most participants (82.4%) expressed interest in further exploring the usage of 3D printing in dentistry (see Figure 4).

## 4 Discussion

In the rapidly advancing field of dentistry, the potential of 3D design and printing to transform dental procedures highlights the need for a knowledgeable and skilled dental workforce. Assessing future dental professionals' readiness to embrace these technologies is crucial. Despite the importance of this assessment, limited research exists on the awareness of 3D applications among upcoming dental practitioners, particularly within the Saudi Arabian context (14). Insights from such assessments could inform educational curricula and professional development strategies. This study addresses this gap by examining the knowledge of 3D design and printing technology among dental students in Saudi Arabia.

The findings from this study revealed a generally moderate to high level of knowledge and awareness among the surveyed dental students regarding the applications and benefits of 3D design and printing in dentistry. Most dental students (61.8%) recognized the benefits of 3D printing, such as enhanced accuracy in implant placement and potential applications in mock surgeries, and over 94.4% knew the advantages of 3D models for precise implant positioning. The findings are consistent with those of Suganna et al., who found that 78.6% of dentists thought that implant placement was the most accurate and least complicated, while 21.4% of dentists thought it was the most difficult and inaccurate procedure (6). Such findings indicate an acceptable level of knowledge that students could have during their studies which can be further developed to provide high-quality services to their patients in the future. However, this study's findings run counter to Jawaharand Maragathavalli’s findings, which indicated that only 8.0% of dentists thought that surgery was becoming more precise and predictable (7).

According to our results, 40.4% of dental students perceived the high cost of equipment as the primary barrier to the widespread adoption of 3D printing in Saudi Arabia. This percentage is somewhat higher than the 27.9% of dentists who identified expensive equipment as the common reason for the lack of 3D technology in dentistry, as reported by Suganna et al. (6). On the other hand, fewer students identified lesser awareness (16.6%) and complexity (4.0%) as individual obstacles limiting the use of 3D technology in the Saudi dental community. This contrasts with a study on 3D technology usage in India, wherein 40% of dentists felt that their lack of knowledge was the main reason, while 15% agreed that the high cost of equipment was the primary factor (7). Notably, 30% of Indian dentists acknowledged multiple barriers, indicating that costs remain a significant barrier (7).

In recent years, 3D printing has been increasingly used across various domains in dentistry, from restorative work, orthodontics, prosthodontics, and oral implantology to instrument manufacturing and dental simulation (15). In this study, the participants demonstrated moderate-to-high levels of awareness regarding the utilization (69.3%) and growing popularity (74.3%) of 3D printing in Saudi dentistry, reflecting a positive reception towards technological innovation among dental students. These findings align with those of Hamed et al. (14), who reported that a significant percentage (70.0%) of dental students in the UAE were aware of advanced imaging technologies like CBCT, indicating a high level of familiarity with modern dental technologies among students. Furthermore, the current study suggests a higher level of awareness compared to the findings of Chandran et al. (16), who reported that 42% of dental students were unaware of the full scope of 3D printing applications in dentistry. The study found multiple variations in knowledge and awareness based on academic level and region. Fifth-year students demonstrated the highest levels of knowledge and awareness, likely due to their advanced stage in education and greater clinical exposure. Dental students in the Eastern regions show higher awareness levels across most questions, likely due to greater resource availability and a stronger focus on technological training and college support.

3D printed drill guides and templates have shown promise in improving the predictability and accuracy of root canal treatments for calcified teeth (17, 18). The technology combines CBCT imaging with CAD-CAM to create custom guides that direct the drill along the planned path to the root canal (19). Recent studies have demonstrated the effectiveness of this approach. For example, one study found that 3D printed guides allowed for predictable treatment of calcified root canals while maximizing the preservation of tooth structure (20). Another *in vitro* study showed that 3D printed guides were accurate and reliable for accessing artificially obliterated teeth and reaching the apical area (21). The growing acceptance of this technology among future and current dental practitioners, as reflected in both the current study and the one by Suganna et al. (6), suggests an increasing awareness of its potential benefits in clinical practice.

The field of orthodontics has also witnessed a remarkable transformation in the manufacturing process of clear aligners, such as those produced by Invisalign, with the advent of 3D printing technology (22). This technology enables the creation of highly precise and customized aligners that fit better and are more effective in achieving the desired tooth movements (22, 23). Advancements in 3D printing technology are now allowing for direct 3D printing of aligners, potentially reducing costs and improving production efficiency and accuracy (23, 24). The new generation of Saudi dentists has shown a strong interest in this technological advancement, as reflected in the awareness among dental students, with 64.0% recognizing the role of 3D printing in creating Invisalign clear aligners. This growing awareness signifies a positive trend towards embracing advanced technologies for improved orthodontic treatment outcomes and professional efficiency.

The primary sources of information about 3D printing were dental colleges (e.g., curriculum, lecturers, online materials provided by the college), seminars and professional presentations (e.g., conferences, online webinars), emphasizing the critical role of academic institutions in disseminating knowledge about emerging technologies. The high interest (82.4%) in further exploring 3D printing applications suggests an openness among students to embrace advanced training and presents an opportunity for dental schools to enhance their curricula. This interest can be leveraged by hands-on training modules, investments in 3D printing equipment, and organizing workshops with industry experts, echoing the optimistic findings from Acharya et al., where a significant interest (98.9%) in 3D printing was also observed among dental professionals (25).

The current study's findings should be interpreted with caution due to several limitations. First, the use of an online cross-sectional, questionnaire-based study design is susceptible to participation and recall bias. Second, the reliance on online sampling through social media may have introduced sampling bias. This means that students who are more interested in the topic or more active online are more likely to participate. Third, the gender imbalance among our participants with a higher proportion of female participants (63.1%) might influence the response frequency. Lastly, the survey outcomes were based on student self-reporting, which may not accurately reflect their actual behaviour in universities' clinics. This is because students may be more likely to report having good knowledge even when they do not. Therefore, the study findings cannot be generalized to other regions or assumed to be true for dental students in countries other than Saudi Arabia.

## 5 Conclusion

The study showed that Saudi Arabian dental students possess satisfactory knowledge and awareness of 3D printing and design, including its benefits, applications, and challenges. To maximize its utilization, improving the accessibility and affordability of 3D printing for students is essential, given their expressed interest in exploring its applications further within dentistry. Integrating 3D design and printing into the curriculum and hosting seminars and workshops are essential for enhancing student proficiency in this transformative technology and preparing them for its future implementation.

## Data Availability

The raw data supporting the conclusions of this article will be made available by the authors, without undue reservation.
