# Supplementary material for: Evaluating knowledge and awareness of 3D design and printing among dental students in Saudi Arabia: a cross-sectional study
Source: Front Dent Med. 2024 Nov 25;5:1466393. doi: 10.3389/fdmed.2024.1466393 (PMC11797805; doi:10.3389/fdmed.2024.1466393)
Supplement: Supplementary file 1 [file Table1.docx]

Supplementary Material

**Table S1:** names of the included universities.

| **University Name** | **Frequency** |
| --- | --- |
| College of Dentistry, Imam Abdulrahman Bin Faisal University | 86 |
| College of Dentistry, Taiba University | 52 |
| College of Dentistry, Al Zulfi - Majma'ah University | 44 |
| College of Dentistry, Hail University | 40 |
| College of Dentistry, Al Jouf University | 34 |
| College of Dentistry, Prince Sattam Bin Abdulaziz | 21 |
| College of Dentistry, King Saud Ibn Abdulaziz University for health sciences | 17 |
| College of Dentistry, King Saud University | 11 |
| College of Dentistry, King Abdulaziz University | 10 |
| Collage of Dentistry, King Faisal University | 10 |
| College of Dentistry, Princes Noura Bint Abdulrahman | 9 |
| College of Dentistry, Najran University | 8 |
| College of Dentistry, Qassim University | 7 |
| College of Dentistry, Dar Al Uloom University | 6 |
| College of Dentistry, Taif University | 5 |
| College of Dentistry, Mustaqbal University | 3 |
| College of Dentistry, Umm Al Qura University | 2 |
| College of Dentistry, Alrass – Qassim University | 2 |
| College of Dentistry, Vision College | 2 |
| College of Dentistry, Jazan University | 2 |
| College of Dentistry, Riyadh Elm University | 2 |
| College of Dentistry, Ibn Sina National College for medical studies | 1 |
| **Total** | **374** |
